# A novel intravascular navigational ultrasound system for transjugular intrahepatic portosystemic shunt procedures

**DOI:** 10.1186/s42155-025-00642-y

**Published:** 2025-12-31

**Authors:** Lei Xiao, Guanqiang Li, Bo Hu, Ming Chen, Yuan Sun, Xicheng Zhang, Xiaohua Jian, Xianchen Huang

**Affiliations:** 1 Department of Vascular Surgery and Intervention, The Fourth Affiliated Hospital of Soochow University, Suzhou, China; 2 School of Materials Science and Intelligent Engineering, Nanjing University, Suzhou, China

**Keywords:** Transjugular intrahepatic shunt, Cirrhosis, Navigation, Puncture, Ultrasound

## Abstract

**Background and aims:**

Transjugular intrahepatic portosystemic shunt (TIPS) is an effective method for reducing portal hypertension in patients with decompensated cirrhosis. However, portal vein puncture is associated with a steep learning curve. Conventional “blind” puncture methods are often imprecise, carry a high risk of complications, and require significant radiation exposure. To increase puncture accuracy, we developed an intravascular navigational ultrasound (IVNU) system. This study aimed to evaluate the feasibility, efficacy, and safety of IVNU for portal vein puncture during TIPS procedures.

**Methods:**

In the in vitro experiment, we performed punctures using IVNU in four isolated porcine livers. Subsequently, in the in vivo animal study, eight Bama swine (*Sus scrofa*) were randomly assigned to undergo TIPS using either IVNU (experimental group) or conventional “blind” puncture with the RUPS100 COOK kit (control group).

**Results:**

In our in vitro experiment, the IVNU system successfully punctured each lobe. In our in vivo study, all the procedures successfully established portosystemic shunts. The IVNU group exhibited significantly fewer punctures (1.8 ± 0.4 vs. 4.2 ± 1.1), shorter procedure times (32.5 ± 4.2 min vs. 58.7 ± 6.5 min), shorter fluoroscopy times (8.1 ± 1.3 min vs. 20.4 ± 2.1 min), and lower radiation doses (579.5 ± 45.9 mGy vs. 1305.7 ± 50.4 mGy) than the control group (all *P* < *0.01*). Puncture-related complications were also significantly reduced in the IVNU group.

**Conclusions:**

These findings indicate that IVNU significantly improves portal vein targeting success, reduces the risk of puncture-related complications and radiation exposure, and decreases procedure time, offering clinicians an optimized solution for TIPS creation.

## Lay summary

This study introduces a new ultrasound-guided system called IVNU that makes puncture procedures safer and more accurate. By allowing doctors to see the portal vein and needle in real time, IVNU reduces the number of needle passes, shortens the operation time, and decreases radiation exposure and complications. This technology could significantly improve the safety and efficiency of TIPS.

## Introduction

Portal hypertension, a life-threatening complication of decompensated cirrhosis, is a leading cause of mortality in patients with advanced liver disease worldwide [1]. Transjugular intrahepatic portosystemic shunt (TIPS), introduced by Richter in 1988, remains the cornerstone minimally invasive treatment for reducing portal pressure [2].

The riskiest and most technically demanding step of TIPS is portal vein puncture from the hepatic vein. Conventional “blind” puncture relies on preoperative imaging and operator experience, resulting in a steep learning curve [3]. Beginners (< 40 procedures) often require multiple attempts, sometimes failing after 6–7 h [4], whereas experienced surgeons achieve only ~ 20% first-puncture success [5], which increases the risks of intraperitoneal hemorrhage, subcapsular/intrahepatic hematomas, and biliary injuries [6].

Several guidance methods for portal vein visualization exist, but each has limitations. Direct portography involves percutaneously puncturing the portal vein through the liver to place a catheter. Indirect portography includes techniques such as CO₂ retrograde portography and indirect exposure of the portal vein via the splenic artery or superior mesenteric artery through the femoral artery [7]. Both of them are potentially risky.

A newer method is the use of image fusion to co-register preoperative CT/MRI with intraoperative DSA for 3D guidance, which enables a first-puncture success rate of approximately 60% [8]. However, respiratory motion and liver deformation frequently introduce spatial misalignments of 1–10 mm between the fused images [9, 10]. The recently developed electromagnetic navigation method mitigates these errors but fails in patients with decompensated cirrhosis [11].

To address the above limitations, ultrasound-based guidance has evolved as a promising real-time alternative [12]. Side-firing intravascular ultrasound (IVUS), also known as intracardiac echocardiography (ICE), was originally used for cardiac procedures such as transseptal puncture [13]; this method provides real-time images and reduces radiation compared with blind methods. Nevertheless, inconsistent alignment between the ultrasound plane and needle trajectory increases the puncture risk. Endoscopic ultrasound (EUS) integrates intraluminal imaging with puncture, enabling gastro-portal or direct intrahepatic shunts [14]. However, it carries risks of gastric perforation, intra-abdominal infection, and stent migration [15].

**Fig. 1:**
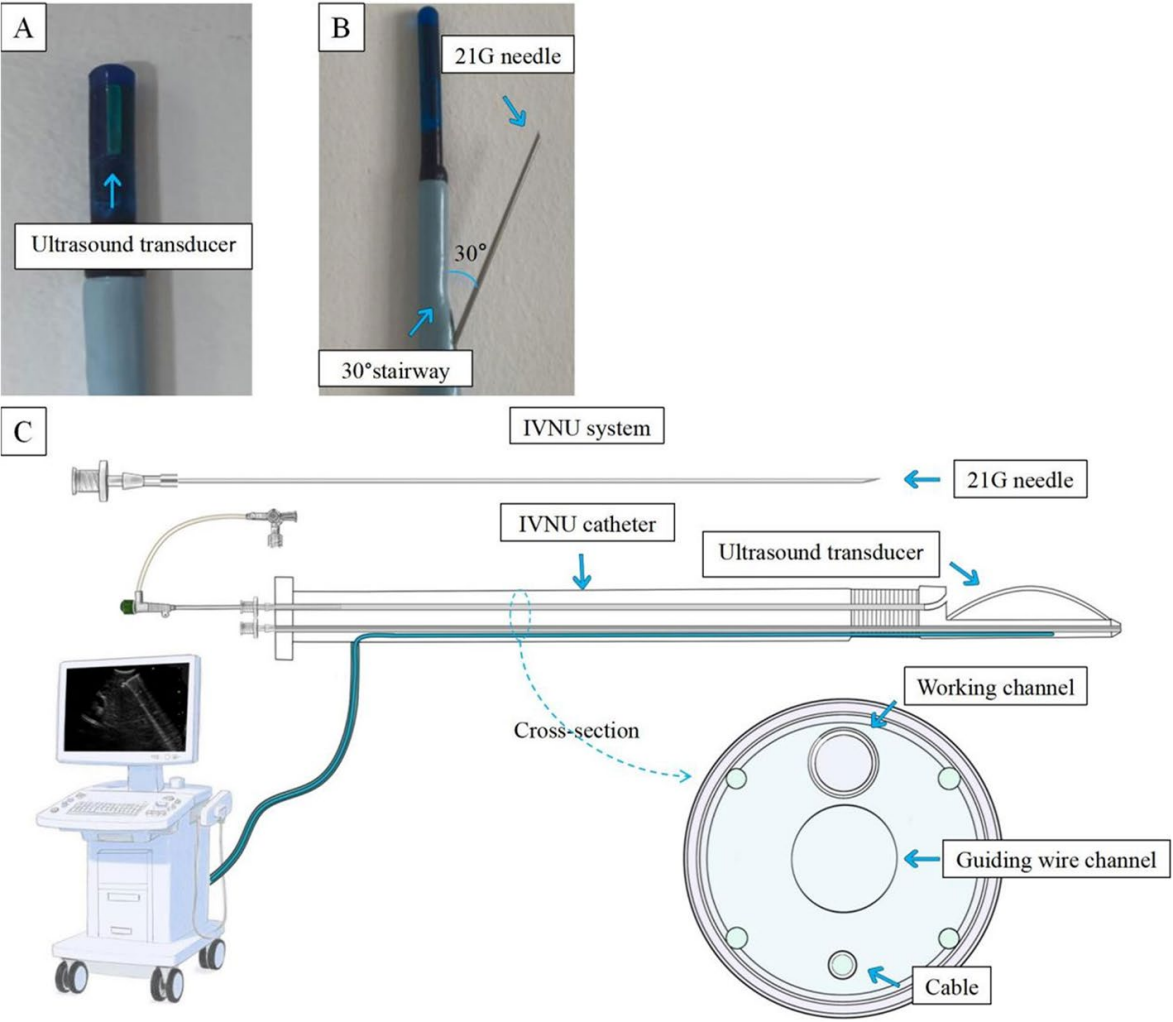
**A** High-frequency ultrasound transducer. **B** The needle outlet is 30° to the central axis. **C** Schematic diagram of the IVNU system

In summary, existing methods have inherent limitations. We hypothesized that real-time synchronization of puncture with portal vein visualization would increase precision and safety. Herein, we developed a steerable intravascular navigational ultrasound (IVNU) system comprising a steerable IVNU catheter, a 21-gauge puncture needle, and an ultrasound console (Fig. 1). The principal contributions of this work to the literature are to develop and demonstrate the functionality of a novel IVNU system, assess its feasibility for TIPS creation through in vitro experiments, and compare its efficacy and safety with those of conventional blind puncture in in vivo porcine models.

## Methods

### Overview of the IVNU catheter

The IVNU catheter features a 12-Fr outer diameter. Its distal tip integrates a high-frequency ultrasound probe (frequency: 10 MHz; axial resolution: 0.1 mm; penetration depth: 8 cm). Proximal to the probe, a 3-cm steerable segment enables 0–90° deflection via a nickel-titanium steering wire. The navigation catheter features a working channel with a diameter of 0.8 mm, which can accommodate a 21G puncture needle. The needle exit point is located 5 mm proximal to the ultrasound transducer tip. There is a fixed-angle platform positioned inferior to the ultrasound probe in the working channel. This ensures that the puncture needle exits at a precise 30-degree angle within the ultrasound imaging plane relative to the central axis, advancing accurately toward the target portal vein. The navigation catheter incorporates a continuous 0.038-inch lumen throughout its length to accommodate a 0.035-inch guidewire. The proximal end of the catheter integrates a steerable control handle and an ultrasound imaging interface port. The system can be seen in Fig. 1.

### General experimental setup

The experiments in this study were performed in an animal laboratory center compliant with ISO 13485, and the operators had performed more than 40 TIPS procedures independently. Livers from 4 adult swine were used for in vitro experiments. For the in vivo animal test, 8 healthy adult Bama swine (*Sus scrofa*, average weight of approximately 50 kg) were used, and they were acclimatized for 7 days preoperatively (temperature 22 ± 2 °C, humidity 55% ± 10%, 12/12 h day/night cycle). The main equipment used included a medical needle tip penetration force tester (CL15811-E, Shanghai Yuanzi Electronic Technology Co., Ltd., China), an IVNU (commissioned by Dingke Medical Technology Suzhou Co., Ltd.), an ultrasound machine (Siemens, Germany), a 21G puncture needle kit (Bait Micro, Shenzhen, China Bait Micro Medical Technology Co., Technology Co., China), a Digital Subtraction Angiography machine (Siemens Healthineers, Germany), an RUPS-100 puncture set (Cook Medical, USA), a 5-mm peripheral intravascular high-pressure balloon dilatation catheter (Priligy, China), a 10-mm × 60-mm bare-metal stent (INNOMED, Suzhou InnoMed Medical Devices Co., Ltd., China), a 0.035-inch hydrophilic guidewire (Eppert, Hunan Eppert Medical Devices Co., Ltd.), a 0.035-inch × 180 cm Amplatz extra-stiff guidewire (Cook Medical, Bloomington, IN, USA), a 5-Fr × 100 cm Multipurpose Angiographic (MPA) catheter (Cook Medical, Bloomington, IN, USA) and contrast medium (Iohexol 350 mg I/mL, GE Healthcare, Chicago, IL, USA).

### Experiment 1: Puncture Resistance Test

The RUPS-100 needle has a relatively large gauge (approximately 18 G), resulting in suboptimal handling precision during puncture [16]. It is particularly unsuitable for patients with excessively hard liver parenchyma. Additionally, its larger bore increases bleeding risk in patients with coagulopathy. To effectively reduce tissue trauma, with an IVNU catheter, we adopted a 21G needle and conducted comparative testing of puncture resistance against the RUPS-100 needle.

Polyurethane-based artificial skin (wall thickness: 1.6 mm) was secured on the test platform. Puncture needles were vertically mounted on a motorized stage and equilibrated in the test environment for 24 h prior to experimentation. Using a penetration force tester (CL15811-E), we measured the peak puncture resistance, defined as the maximum force required for needle penetration through the artificial skin membrane. Three valid punctures were performed with three independent needles. The mean puncture resistance of the 21-gauge needles used in our system was compared against that of the commercially available RUPS-100 needle.

### Experiment 2: in vitro model validation

Four fresh intact porcine livers were immersed in saline. A red pigment-saline solution was continuously perfused into the portal vein using a peristaltic pump, and the portal pressure was maintained at 10 mmHg to simulate physiological hemodynamics [17].

We performed IVNU-guided hepatic-to-portal vein punctures in each of four hepatic lobes: left lateral, left medial, right lateral, and right medial. The navigation catheter was inserted into a target hepatic vein, with real-time ultrasonic guidance facilitating adjustments of position and angulation until the target portal vein aligned with the puncture trajectory. A 21-gauge needle was then advanced through the working channel to penetrate the portal vein. Successful puncture was confirmed by aspiration of red-tinged fluid upon needle core withdrawal. If unsuccessful, the needle was repositioned for repeat attempts. Following successful shunt establishment in one lobe, the procedure was repeated sequentially in the remaining lobes.

### Experiment 3: in vivo animal study

Eight adult Bama swine (mean weight: 50 kg) were utilized in our study and randomized into an experimental group (n = 4) receiving TIPS via the IVNU system and a control group (n = 4) receiving a conventional “blind” puncture method with a RUPS-100 puncture set.

In the experimental group, anesthetized swine received endotracheal intubation with mechanical ventilation and continuous monitoring. Vascular access through right jugular vein puncture was established under ultrasound guidance following disinfection. A 5-Fr MPA catheter was advanced over a 0.035-inch hydrophilic guidewire through the superior vena cava (SVC) and right atrium (RA) into the inferior vena cava (IVC) to locate the hepatic vein ostium. After the 12-Fr long sheath and 180-cm stiff guidewire were exchanged, the IVNU catheter was inserted into the hepatic vein of the right medial lobe. Adjustment of the catheter’s distal bending angle (0–90°) and position enabled ultrasonic visualization of the portal vein. The target portal vein was aligned with the puncture trajectory by controlled catheter rotation and advancement/retraction. A 21-gauge needle was then advanced through the working channel to penetrate the portal vein under continuous ultrasonic guidance. Successful entry was confirmed by aspiration of blood upon needle core withdrawal, supplemented by portal venography. After successful puncture, the IVNU catheter was withdrawn. Following the placement of a 0.018-inch guidewire, a 5-mm balloon catheter was used to dilate the parenchymal tract to create a pathway. An angiographic catheter was then introduced to exchange for a 0.035-inch stiff guidewire. Over the stiff guidewire, a 10 × 60 mm bare metal stent was inserted and deployed in position, followed by postdilation with 8*60 mm balloon angioplasty. Finally, angiography was performed to assess stent patency. This protocol was replicated in the remaining three experimental swine.

For the control group, we similarly advanced the guidewire and catheter into the hepatic vein of the right medial lobe in porcine livers. On the basis of this imaging and operator experience, the angle of the RUPS-100 needle was adjusted for portal vein targeting. The needle was advanced to penetrate the hepatic parenchyma and enter the target vessel. The portal vein was confirmed by blood reflux and venography. Puncture was repeated with the trajectory or curvature modification if no blood refluxed until successful shunting. Following confirmed entry, the hepatic parenchymal tract was dilated, and a bare-metal stent was then deployed to establish the shunt. Final angiography revealed the hepatic vein, portal vein, and intrastent shunt channel. This protocol was replicated in the remaining three Bama swine.

### Study endpoints and statistics

The primary endpoint was successful puncturing, and the secondary endpoint was the incidence rate of complications. All the animals underwent immediate postprocedural euthanasia. To prevent observation bias, interventional operators and complication assessors maintained operational independence during endpoint evaluation.

### Observation endpoints

(1) Puncture procedure time (min): From jugular vein puncture to the puncture needle successfully; (2) Number of puncture attempts: Count of needle passes required for channel creation; (3) Fluoroscopy time (min) and radiation dose (μGy): Cumulative metrics during our procedure; (4) Procedure-related complications: ① Intraperitoneal hemorrhage: Assessed via laparotomy for active bleeding or significant hemoperitoneum, including subcapsular hepatic hematoma evaluation; ② Pericardial hemorrhage: Documented by direct inspection of blood accumulation in the pericardial cavity during thoracotomy; ③ Cardiac arrhythmias: Defined as sustained ventricular premature complexes or ventricular tachycardia detected on continuous ECG monitoring; ④ Adjacent organ injury: Systematic examination for diaphragmatic perforation, biliary tract damage, gastric/intestinal injury, or abdominal wall trauma during necropsy.

Statistical analyses were performed using SPSS 23.0 (IBM Corp.). All the raw data were verified and calibrated prior to analysis. Continuous variables are expressed as the mean ± standard deviation (mean ± SD), whereas categorical variables are presented as frequencies (%). The normality of the distribution was assessed using Shapiro–Wilk tests, which are appropriate for small sample sizes. For normally distributed data with homogeneous variance (confirmed by Levene's test), one-way ANOVA was applied; nonnormally distributed data or heterogeneous variance data were analyzed using the Mann–Whitney U test (two-independent-sample rank-sum test). Statistical significance was defined as p < 0.05.

## Results

### Experiment 1: Puncture Resistance Test

In this study, we employed a 21G needle in the IVNU system. By comparing the average puncture force of the 21G needle with that of the conventional RUPS-100 Puncture Kit in synthetic tissue models, we found that the average puncture force of the RUPS-100 (5.373 N) was significantly greater than that of the 21G needle (3.834 N), representing a difference of approximately 40.2% (see Table 1).

**Table 1:** Mean penetration resistance test

Product	Test 1 N	Test 2 N	Test 3 N	Mean ± SD N	CV
21G	3.856	3.816	3.830	3.830 ± 0.020	0.520%
RUPS100	5.547	5.299	5.272	5.370 ± 0.150	2.790%

### Experiment 2: in vitro model validation

**Fig. 2:**
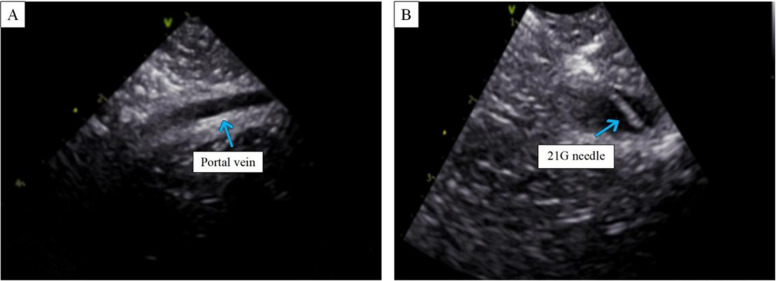
Ultrasound images of the liver and portal vein in vitro. **A** An observation of the target portal vein using IVNU. **B** An observation of the 21G puncture needle accessing the target portal vein

The IVNU system achieved 16 successful portal vein punctures out of 36 attempts across 16 hepatic lobes from four isolated porcine livers (success rate: 44%; Table 2). Real-time IVNU imaging was used to visualize hepatic and portal venous anatomy (Fig. 2). Key anatomical observations revealed distinct vascular characteristics influencing procedural outcomes: The left lateral lobe hepatic veins demonstrated a narrow lumina (5.5 ± 0.8 mm in diameter; Table 2) with intricate branching patterns and morphological features associated with elevated puncture failure rates. In contrast, the right lateral and medial lobes share a common vascular trunk characterized by straight trajectories and short-diameter vessels and anatomical configurations conducive to enhanced puncture efficiency. Quantitative analysis corroborated this disparity, with the right medial lobe requiring significantly fewer puncture attempts (1.3 ± 0.4 punctures/liver lobe) than the other hepatic segments did (P < 0.05), indicating a direct correlation between anatomical architecture and procedural success rates.

**Table 2:** Hepatic vein anatomical data and number of punctures

Liver Lobe	Puncture Attempts (n)	Trunk Diameter (mm)	Branch Diameter (mm)	Success Rate (%)
Left lateral	3.0 ± 0.7	5.5 ± 0.8	6.0 ± 1.0	33
Left medial	2.8 ± 1.1	6.0 ± 1.0	5.0 ± 1.0	36
Right lateral	2.0 ± 0.7	10.0 ± 2.0	10.0 ± 2.0	50
Right medial	1.3 ± 0.4	7.5 ± 1.2	6.0 ± 1.0	80

An IVNU catheter was inserted into the inferior vena cava of the swine to observe the diameters of the trunk and branches of the hepatic vein in each hepatic lobe, and then an IVNU catheter was inserted into each hepatic lobe, and the portal vein was punctured until blood was withdrawn; the number of punctures required for successful puncture was recorded.

#### Experiment 3: in vivo animal experiments

**Fig. 3:**
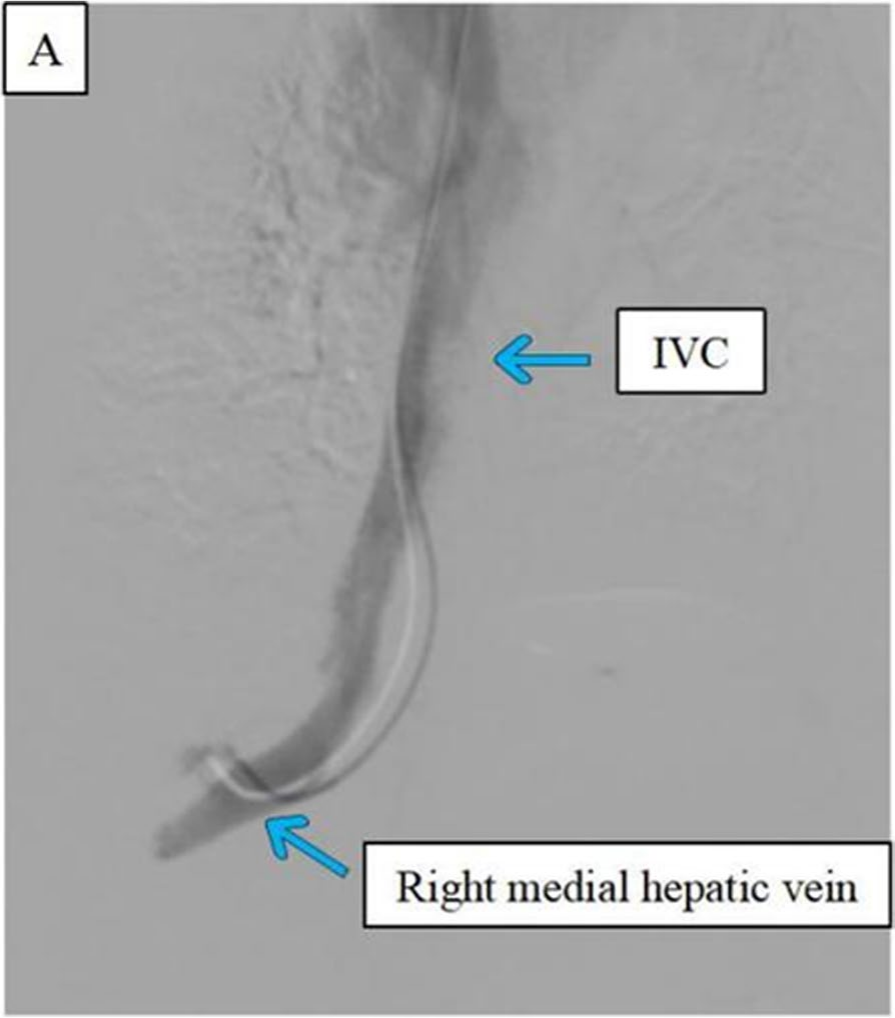
**A** Visualization of a porcine right medial hepatic vein

**Fig. 4:**
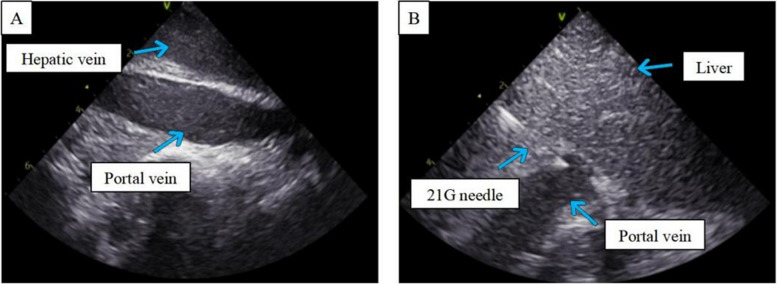
Ultrasound images captured in vivo with IVNU. **A** Right medial hepatic vein. **B** Right medial hepatic vein punctured by an IVNU-guided 21G needle

**Fig. 5:**
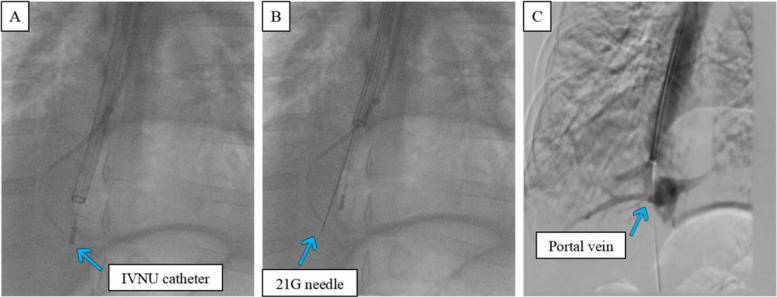
**A** Entry of the IVNU catheter into the right medial hepatic vein. **B** The 21G puncture needle accessing the portal vein. **C**: Portal vein venography

**Fig. 6:**
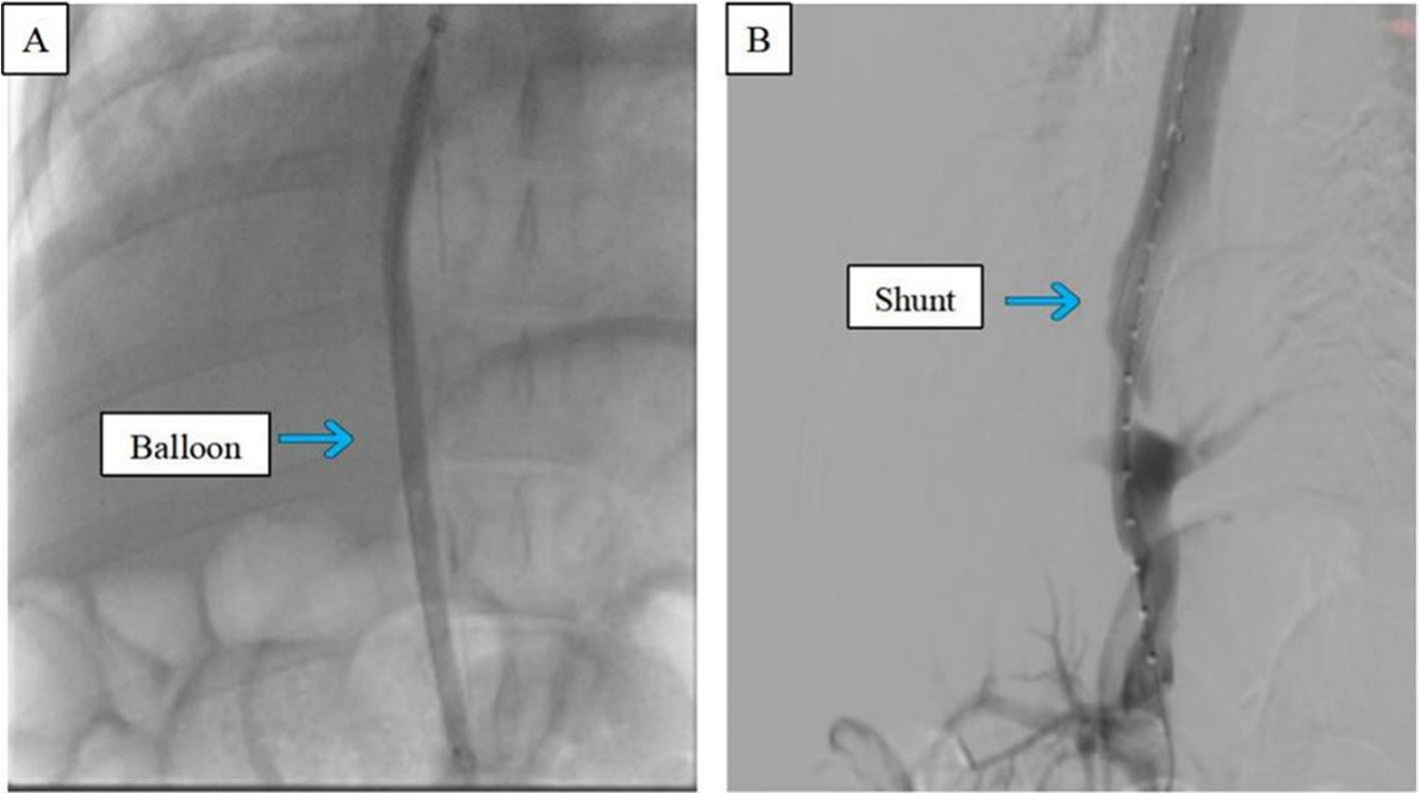
**A** Balloon dilatation. **B** Portal vein venography after stent placement

As shown in Fig. 3, we consistently selected the hepatic veins of the right medial lobe, irrespective of whether they were in the IVNU group or the blind puncture group. The images in Fig. 4 subsequently demonstrate a clear portal vein image obtained via an IVNU ultrasound probe from the hepatic vein perspective, followed by successful portal vein puncture using a 21-gauge needle. DSA imaging was employed to observe the IVNU catheter and needle position, with subsequent portal venographic verification of puncture success (Fig. 5). Finally, as shown in Fig. 6, we dilated the puncture tract by balloon and created a portosystemic shunt by stent deployment, with subsequent venographic confirmation of hemodynamic patency.

**Fig. 7:**
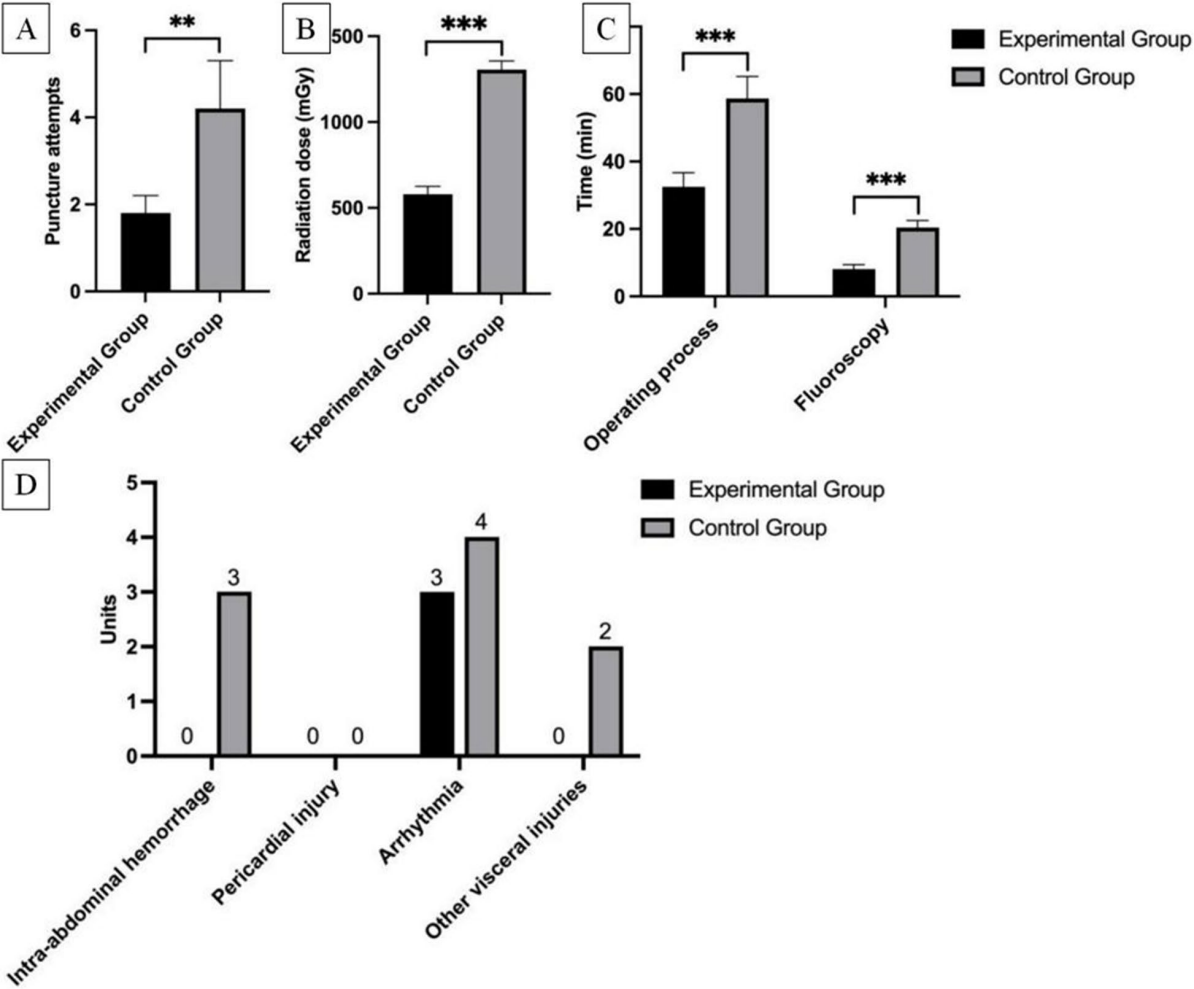
**A** Comparison of the average number of punctures. **B** Comparison of the radiation dose. **C** Comparison of the duration of the operating process in general and the fluoroscopy in particular. D Comparison of the number of complications

During transjugular intrahepatic portosystemic shunt (TIPS) procedures in Bama swine, both the IVNU experimental group and the conventional “blind” control group successfully established functional shunt channels. In the IVNU group, single-pass portal vein puncture was achieved in 50% of the attempts, whereas the success rate was 23.5% in the blind puncture group. The IVNU group (n = 4) demonstrated significantly superior outcomes. The mean number of needle passes (1.8 ± 0.4) was 57.1% lower than that in the blind puncture group (4.2 ± 1.1, *p* < *0.001*). Significant reductions were also observed in the fluoroscopy time (8.1 ± 1.3 min vs. 20.4 ± 2.1 min; −60.3%) and radiation dose (579.5 ± 45.9 mGy vs. 1305.7 ± 50.4 mGy; −55.6%), both *P* < *0.01*. The complication rates differed: the IVNU group had 3 cases of cardiac arrhythmias but no incidences of intraperitoneal hemorrhage, pericardial hemorrhage, or adjacent organ injury; in contrast, the control group exhibited higher incidences of intraperitoneal hemorrhage (3 cases), cardiac arrhythmias (4 cases), and adjacent organ injury (2 cases). These quantitative outcomes demonstrate that the IVNU system provides superior targeting precision, a shorter procedure duration, substantially reduced radiation exposure, and fewer complications than conventional landmark-based puncture does. These detailed data are shown in Fig. 7.

## Discussion

Since Joseph Roesch's pioneering animal experiments on transjugular intrahepatic portosystemic shunt (TIPS) in 1969 [18], this procedure has made progress in multiple aspects. However, the current literature indicates a steep learning curve for safe TIPS execution. To improve puncture accuracy and further reduce fatal procedure-related complications, more than 150 procedures are needed [19]. This difficulty exacerbates complex anatomies, including portal vein anatomical abnormalities, portal vein thrombosis, and Budd–Chiari syndrome. For instance, even in patients with decompensated cirrhosis without portal vein thrombosis or Budd-Chiari syndrome, conventional blind TIPS procedures achieve technical success rates of only about 94% and frequently require multiple needle passes [14, 20]. With the increasing prevalence of global cirrhosis and consequent thrombotic comorbidities, the limitations of conventional “blind” puncture are becoming increasingly evident.

To improve the accuracy of portal vein puncture during TIPS procedures, numerous assisted puncture techniques have emerged. Clinically, direct or indirect portography is typically used to guide punctures. Direct portal venography via splenic or paraumbilical puncture [21], or percutaneous ultrasound-guided access [22], enables real-time visualization but relies on 2D DSA, with vessel shifts and limited angle-prolonging procedures. Percutaneous transhepatic catheterization with a target catheter addresses displacement [23] but risks subcutaneous hemorrhage, subcapsular hematoma, and hemoperitoneum. CO_2_ angiography reduces radiation but lacks real-time imaging, potentially worsening risks such as air embolism or poor visualization leading to mispuncture [24]. Similarly, splenic artery or superior mesenteric artery angiography via femoral access often fails in patients with decompensated cirrhosis. While reports in the literature indicate that the aforementioned techniques achieve portal vein puncture in TIPS procedures with an average of 3 needle passes [25], these methods introduce additional puncture-related trauma. Moreover, the acquired two-dimensional vascular images provide limited guidance for precise needle positioning.

Given the limitations of these ancillary techniques, a nonnegligible proportion of us have shifted their focus toward image fusion technology for puncture guidance. This approach reduces the procedural complexity of the procedure. In current clinical practice, CT/MR images are typically integrated with portography [26], but this technique is universally hampered by the 'CT/MR-to-body divergence' effect [27]. This results in registration discrepancies between the fused images and real-time DSA, which paradoxically introduces targeting deviations during needle advancement. Although Huibin Shi et al. reduced registration errors using a modified cone-beam CT dual-phase 3D vascular image fusion technique [28], inherent limitations from hepatic displacement and respiratory motion persist, making residual matching inaccuracies unavoidable in practical applications. Advancing this field, Medis Medical has developed the Imedis9000 Puncture Guidance System [29]. This technology integrates CT/MR images with ultrasound, incorporates magnetic navigation, and enables real-time external ultrasound guidance for portal vein puncture. However, this device requires cumbersome external ultrasound coordination during puncture.

With advancements in imaging technology, intraluminal ultrasound has been innovatively applied to TIPS procedures. First, Rai, P reported EUS-guided TIPS creation. Using an echoendoscope [30], the authors performed transgastric puncture of the inferior vena cava (IVC) and portal vein to establish a shunt between them. This technique has significant limitations: it increases procedural trauma and infection risk from gastric wall puncture, and endoscopic ultrasound (EUS) by nature cannot be deployed intravascularly.

Side-firing IVUS and ICE represent the current intravascular ultrasound modalities applicable in TIPS procedures [31]. A growing body of literature documents their clinical deployment, with their critical advantage lying in direct intraluminal visualization of perivascular anatomy. Side-firing IVUS is typically known as intracardiac echo ICE; its catheter's imaging plane runs parallel to the device axis, enabling real-time puncture guidance. However, conventional ICE catheters have an imaging plane oriented nonparallel to the puncture needle trajectory. This misalignment paradoxically prolongs procedural duration and delivers suboptimal clinical benefit. There is therefore a clinical imperative for technologies enabling precise puncture with a shortened learning process, resulting in increased cost-effectiveness for patients.

Building upon the technical foundations of EUS and ICE, we invented the IVNU system. This system includes a steerable navigation catheter, a 21-gauge ultrafine puncture needle, and an ultrasound main unit. The ultrasound probe at the catheter tip can enter the hepatic vein to visualize the portal vein in real time, while the needle channel within the catheter allows a 21G puncture needle to pass through. In in vitro experiments, our 21G puncture needle resulted in a 28.7% reduction in the average puncture resistance force compared with that of the RUPS100, effectively lowering the risks of vascular injury and surgical complications. By design, our IVNU can precisely locate the portal vein and guide punctures, shortening the learning process and providing a safer, more efficient technical approach for TIPS procedures.

This study demonstrates the feasibility, safety, and efficacy of IVNU-guided puncture for TIPS creation through ex vivo porcine liver models and in vivo animal experiments. In the ex vivo experiments, we visualized the portal vein via the IVNU catheter positioned within the hepatic vein and manipulated the catheter to achieve targeted portal vein puncture. A total of 36 puncture attempts were performed, with a success rate of 44%. Twenty attempts failed to access the portal vein because of hepatic displacement during needle advancement, causing deviation of the portal vein from the intended puncture trajectory. This phenomenon occurs because ex vivo livers lack stabilizing supporting ligaments, permitting greater freedom of movement. Furthermore, compared with other liver lobes, our ex vivo liver model demonstrated the highest success rate (80%) in the right medial lobe of the porcine liver. Consequently, we selected this hepatic segment for puncture in subsequent in vivo experiments [32].

During in vivo animal experiments, we successfully achieved precise portal vein puncture. With the IVNU catheter tip positioned within the hepatic vein, significantly sharper ultrasound images of anatomical delineation were obtained than under ex vivo conditions. In ex vivo porcine livers, air entrapment within the parenchyma compromises ultrasound image quality during experimental procedures. We subsequently manipulated the catheter to visualize the portal vein’s caliber and course. After the target portal vein was identified and confirmed, the needle was advanced toward the vessel under real-time guidance, which resulted in a single-puncture success rate of 50%. During the puncture attempts, we observed mild parenchymal displacement in the in vivo porcine livers, which subsequently led to puncture failure. Nevertheless, this displacement was significantly reduced compared with that documented in ex vivo experiments. In our experiments, the portal vein diameter in the right medial lobe was generally 5–6 mm, which is obviously smaller than the 10–12 mm typically observed in cirrhotic patients. Thus, applying this technique in human subjects could further increase the puncture success rate. Additionally, our IVNU system enables real-time quantification of the needle trajectory length, facilitating precise stent size selection, and allows postdeployment evaluation of the stent lumen to promptly exclude compression, kinking, or other potential complications.

In the in vivo animal experiments, compared with the control group, the IVNU group demonstrated significant advantages. Compared with the blind puncture group, the IVNU group demonstrated a greater puncture success rate (57.1% vs. 23.5%, *p* < *0.01*), fewer average needle passes (1.8 ± 0.4 vs. 4.2 ± 1.1, *p* < *0.01*), and a markedly shorter procedural duration (32.5 ± 4.2 min vs. 58.7 ± 6.5 min, *p* < *0.001*); compared with the blind puncture group, the IVNU utilization group had shorter fluoroscopy times (8.1 ± 1.3 min vs. 20.4 ± 2.1 min, *p* < *0.001*) and substantially lower radiation doses (579.5 ± 45.9 mGy vs. 1305.7 ± 50.4 mGy, *p* < *0.001*).

Through IVNU-guided puncture, continuous real-time visualization of the advancing needle prevents accidental injury to extrahepatic organs. This study revealed fewer complications in the IVNU group than in the control group, which aligns with Maria et al.’s published findings on ICE catheter-assisted portal vein puncture. Furthermore, compared with published studies, our IVNU group demonstrated a shorter procedure duration [33]. While the ICE-guided approach (Ramaswamy et al.) is limited by the difficulty in aligning the independent imaging plane with the needle path, our IVNU system overcomes this limitation by integrating a fixed-angle needle guide adjacent to the transducer. This design guarantees that needle advancement occurs within the imaging plane, reducing the procedure time. Thus, our IVNU system demonstrates distinct advantages over conventional blind puncture methods by enhancing TIPS targeting accuracy, reducing puncture-related complications, and lowering procedural complexity.

However, this study has certain limitations. First, the product used in this experiment was an initial prototype with certain manufacturing imperfections, which may have somewhat compromised the experimental outcomes. Second, the relatively small sample size (only four subjects per group) resulted in a statistically nonsignificant difference in complication rates despite the observed numerical trends. Finally, because we exclusively used healthy porcine livers in our animal experiments and did not include a cirrhosis model, we were unable to fully replicate the TIPS procedures performed in human cirrhosis patients. Therefore, subsequent research will focus on refining the design details of the IVNU system and conducting adequately powered studies in cirrhotic models.

## Conclusions

In this study, compared with conventional blind puncture methods, the novel intravascular navigation ultrasound (IVNU) system enables clear visualization of portal venous anatomy, provides real-time puncture guidance, achieves superior success rates, reduces radiation exposure, and minimizes puncture-related complications during transjugular intrahepatic portosystemic shunt (TIPS) procedures. This technology has the potential to substantially reduce the technical demands of TIPS procedures, shorten the learning process, and expand accessibility, enabling more clinicians to safely adopt this complex technique.

## Data Availability

Not applicable.
